# Effect of short-term heart rate variability biofeedback on long-term abstinence in alcohol dependent patients – a one-year follow-up

**DOI:** 10.1186/s12888-017-1480-2

**Published:** 2017-09-06

**Authors:** Ana Isabel Penzlin, Kristian Barlinn, Ben Min-Woo Illigens, Kerstin Weidner, Martin Siepmann, Timo Siepmann

**Affiliations:** 1 Treatment Center for Addiction Disorders, Heidehof Hospital, Weinböhla, Germany; 2 Department of Neurology, University Hospital Carl Gustav Carus, Technische Universität Dresden, Fetscherstr. 74, 01307 Dresden, Germany; 3 Department of Neurology, Beth Israel Deaconess Medical Center, Harvard Medical School, Boston, MA USA; 4 Department of Psychotherapy and Psychosomatic Medicine, University Hospital Carl Gustav Carus, Technische Universität Dresden, Dresden, Germany

**Keywords:** HRV, Heart rate variability, Biofeedback, Autonomic, Abstinence, Alcohol addiction, Craving, Rehabilitation, Survey, Relapse

## Abstract

**Background:**

A randomized controlled study (RCT) recently showed that short-term heart rate variability (HRV) biofeedback in addition to standard rehabilitation care for alcohol dependence can reduce craving, anxiety and improve cardiovascular autonomic function. In this one-year follow-up study we aimed to explore whether completion of 2-week HRV-Biofeedback training is associated with long-term abstinence. Furthermore, we sought to identify potential predictors of post-treatment abstinence.

**Methods:**

We conducted a survey on abstinence in patients with alcohol dependence 1 year after completion of an RCT comparing HRV-biofeedback in addition to inpatient rehabilitation treatment alone (controls). Abstinence rates were compared and analysed for association with demographic data as well as psychometric and autonomic cardiac assessment before and after completion of the biofeedback training using bivariate and multivariate regression analyses.

**Results:**

Out of 48 patients who participated in the RCT, 27 patients (9 females, ages 42.9 ± 8.6, mean ± SD) completed our one-year follow-up. When including in the analysis only patients who completed follow-up, the rate of abstinence tended to be higher in patients who underwent HRV-biofeedback 1 year earlier compared to those who received rehabilitative treatment alone (66.7% vs 50%, p = ns). This non-significant trend was also observed in the intention-to-treat analysis where patients who did not participate in the follow-up were assumed to have relapsed (46,7% biofeedback vs. 33.3% controls, p = ns). Neither cardiac autonomic function nor psychometric variables were associated with abstinence 1 year after HRV-biofeedback.

**Conclusion:**

Our follow-up study provide a first indication of possible increase in long-term abstinence after HRV-biofeedback for alcohol dependence in addition to rehabilitation.

**Trial registration:**

The original randomized controlled trial was registered in the German Clinical Trials Register (DRKS00004618). This one-year follow-up survey has not been registered.

## Background

Alcohol dependence is a major global public health problem which affects 5.1% of the global population and causes up to 3.3 million deaths per year, the majority of those being related to cardiovascular diseases [1]. Although integrative multimodal acute and rehabilitative treatment regimens have been widely established to reduce the disease burden related to alcohol dependence, malcompliance and low rates of adherence to treatment are frequently compromising success of these therapies [2, 3]. Moreover, even after completion of rehabilitation relapse poses a major problem. Major predictors of post-treatment relapse have been identified in large observational studies, including substance use patterns prior to treatment, psychiatric comorbidities, social and psychological characteristics as well as craving [4–9].

We recently showed in a randomized controlled study that heart rate variability (HRV) biofeedback in addition to standard rehabilitation care for alcohol dependence can reduce craving and anxiety more effectively than rehabilitative treatment alone [10]. In this study, we also observed improvement in cardiac autonomic and neurovascular function in patients undergoing biofeedback possibly mediated by counterbalancing a chronic shift toward an increased sympathetic and decreased parasympathetic tone. However, our short-term follow-up data did not answer the question whether the observed improvements in psychometric and cardiac autonomic endpoints translate into reduced risk of post-treatment relapse.

In this follow-up study we aimed to determine the relapse rate 1 year after HRV-biofeedback training and integrative inpatient rehabilitative treatment in order to explore potential treatment effects and acquire first long-term data that could form a basis for confirmatory research in large study populations. Furthermore, we sought to identify predictors of abstinence following combined rehabilitative and HRV-biofeedback treatment.

## Methods

### Study design and population

This is a one-year follow-up survey study after a randomized controlled trial on the effects of HRV-biofeedback on cardiac autonomic function assessed via time and frequency domain parameters of HRV, autonomic neurovascular function assessed via laser Doppler flowmetry of cutaneous blood flow after sympathetic stimulation as well as craving, anxiety and depressive symptoms evaluated using psychometric tests. These techniques were reported in detail elsewhere [10]. Briefly, male and female patients undergoing inpatient rehabilitation treatment for alcohol use disorder received either HRV-biofeedback in addition to standard rehabilitative care or standard rehabilitative care only. The study intervention comprised application of a validated HRV-biofeedback system (StressPilot™; BioSign, Ottenhofen, Germany) with continuous measurement and real-time visualization of HRV. Study subjects were instructed to breathe at a given frequency of six cycles per minute to increase the parasympathetic tone and thereby HRV. Patients in the HRV-biofeedback group underwent three 20-min sessions of HRV-biofeedback training per week over 2 weeks whereas control patients did not undergo biofeedback. Psychometric testing, and assessment of neurovascular and autonomic cardiac function were undertaken before the beginning of the first biofeedback session, immediately after completion of the last biofeedback session as well as 3 and 6 weeks afterwards.

To perform a one-year follow-up assessment of abstinence, study participants were contacted by mail 1 year after discharge from rehabilitative therapy after having given written permission to be contacted during inpatient treatment. They were asked to answer a standard questionnaire about their social situation, alcohol and drugs consumption, as well as need for further institutional treatment in the period of 12 months after discharge. Abstinence from alcohol and drugs was considered no consumption during 12 months after discharge from our clinic. We extracted demographic characteristics in subjects who had undergone HRV-biofeedback in addition to rehabilitation 1 year earlier and those who had received rehabilitative treatment alone from the original dataset.

### Comparison of abstinence rates

Abstinence rates 1 year after discharge from the rehabilitative therapy were evaluated applying standard criteria of the German Society for Addiction Research and Addiction Treatment [11]. Individuals who have not consumed any alcohol since discharge from the inpatient treatment were considered abstinent whereas any alcohol consumption post-rehabilitation was defined relapse. In a first analysis we included all patients who participated in the original study protocol and returned our survey 1 year later to compare rates of abstinence in patients that had been allocated to the HRV-biofeedback group and those who had undergone rehabilitative treatment alone during the RCT. We then went on and repeated the comparative analysis applying an intention-to-treat approach, including the entire study population of the RCT irrespective of whether they have participated in the one-year follow-up. In this more conservative analysis, non-responders were considered to have relapsed.

### Analysis of factors related to abstinence

In order to define individual factors related to abstinence we compared patients who reported being abstinent 1 year post-intervention and those who reported relapse in terms of demographic characteristics as well as psychometric measurements and parameters of the autonomic cardiac function. These analyses were performed in patients who completed follow-up. We included in our analyses psychometric scores at baseline and those obtained immediately following the final HRV-biofeedback training session (post-biofeedback or post-observation period for patients in the control group). Psychometric scores were obtained using the Obsessive Compulsive Drinking Scale, as measure of craving as well as the subscales Anxiety and Depression from the Symptom Checklist-90, as measures of anxiety and depression. Measures of the cardiac autonomic function comprised the time-domain parameter coefficient of variation of R-R intervals (CVNN) and frequency-domain parameters high frequency (HF), low frequency (LF) and total power (TP) at baseline and immediately post-biofeedback or observation period. In order to improve our understanding of the association between applied treatments, and sustained abstinence, this analysis was undertaken in both the biofeedback and the control group.

We then went on to identify specific predictors of abstinence after rehabilitative inpatient care by relating baseline data extracted from the original dataset of the randomized controlled trial to data from the present survey. We conducted these analyses separately for control patients and those who had received HRV-biofeedback. In order to identify any specific changes in biological or psychological characteristics due to HRV-biofeedback which relate to sustained abstinence, analyses were repeated using data on patient characteristics and outcomes obtained immediately after completion of the biofeedback intervention, the duration of the control period, respectively.

### Statistical analysis

Statistical analyses were performed using SPSS ® 21 (IBM, Armonk, NY, USA). Dichotomous data were compared using Ӽ^2^ test in patients who completed follow-up. Continuous data on study population characteristics were compared between patients who had received HRV-biofeedback in addition to rehabilitation and those who had undergone rehabilitative treatment only using Student’s t–test or Mann-Whitney U test, according to distribution. The same tests were used to compare study population characteristics and post-biofeedback outcomes between patients who reported abstinence after 1 year and those who had relapse. These analyses were first undertaken in those patients that have participated in the follow-up assessment excluding those that have not returned the survey questionnaire. The same tests were then applied to undertake an intention-to-treat analysis in the entire study population, considering those who have not participated in the one-year follow-up to have relapsed. Comparisons between patients with and without achievement of on-year abstinence were performed separately for the biofeedback and the control group. Alpha level for statistical significance was set to 0.05. Bivariate logistic regression analyses were undertaken to identify possible predictor variables among demographic and outcome variables with respect to one-year abstinence in patients who completed follow-up. Those variables that emerged as predictors of abstinence were included in multivariate models with adjustment for demographic characteristics. Unstandardized B-coefficients (ß) and *p*-values were computed.

## Results

### Demographic characteristics

Among 48 study participants who have been contacted by mail, 27 responded and completed the questionnaire (56.2%; 15 biofeedback and 12 control patients, 18 males and 9 females, ages 42.9 ± 8.6, mean ± SD). Demographic characteristics of patients who completed follow-up are shown in Table 1. Within this population, there were no differences in age, gender, tobacco use, neuropathy and number of cases of liver disease between patients who have undergone biofeedback in addition to rehabilitative treatment and those who have undergone rehabilitation only. (Table 1) Demographic characteristics of the study population which consists of all patients included in the intention-to-treat analysis are reported elsewhere and have been additionally amended to Table 1 [10].

**Table 1:** Demographic Characteristics

	Patients who completed follow-up			Intention-to-treat		
	HRV Biofeedback (*n* = 15)	Control (*n* = 12)	*p*-value	HRV Biofeedback (*n* = 24)	Control (*n* = 24)	*p*-value
Age (years)	41.2 ± 8	43.6 ± 9	0.74	40 ± 7	44 ± 8	0.06
Gender (%)	53.3 m, 46.7 f	83.3 m, 16.7 f	0.09	70.8 m, 29.2 f	70.8 m, 29.2 f	0.09
Smoking (%)	80	75	0.75	79	75	0.36
Comorbidities						
• Neuropathy (%)	26.7	0	0.05	33.3	16.7	0.09
• Hepatic steatosis (%)	20	50	0.05	20.8	37.5	0.07

Analyses of demographic data showed no differences in size, weight, gender, tobacco use and comorbidities between patients who have undergone HRV-biofeedback one year earlier and those who have received rehabilitation only. This was true when both, only patients who completed follow-up or all participants of the interventional randomized controlled trial (Intention-to-treat) were included in the analyses

*m* male*, f* female

### Abstinence

**Fig. 1:**
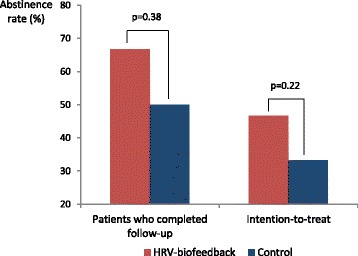
Abstinence rate one year after discharge from the rehabilitative therapy. The bar graph shows the rates of one-year abstinence in each study arm (HRV-biofeedback and control). Results are separately displayed for patients who completed follow-up and the entire population (intention-to-treat). A non-significant trend toward increased abstinence one year post-HRV-biofeedback (*red bars*) compared with control patients who have not undergone the intervention (*blue bars*) was observed when only complete cases were included. This was also true in the intention-to-treat analysis where patients who have not completed follow-up were considered to have relapsed was performed

When including only patients who completed follow-up in the analyses, abstinence rates tended to be higher in those who had undergone HRV-biofeedback in addition to rehabilitation compared to those who received rehabilitative treatment alone. Similarly, the intention-to-treat analysis, where patients who have not completed follow-up were considered to have relapsed, also showed a trend toward higher abstinence rates among patients who had undergone HRV-biofeedback than those who underwent rehabilitation alone (Fig. 1).

### Characteristics of abstinent patients vs. relapse patients

In order to identify factors important to abstinence we compared demographic characteristics as well as outcome data on psychometric tests and autonomic cardiac function at baseline and immediately after completion of the RCT between patients who showed abstinence 1 year after completion of the trial and those who had relapse. As shown in Table 2, none of the included study population characteristics were different between abstinent patients and those who had relapse. This was true for both, the biofeedback group and the control group.

**Table 2:** Characteristics comparison between abstinent and relapsed patients from HRV-biofeedback and control groups

	HRV-biofeedback		*p*-value	Control		*p*-value
	Abstinent (*n*=10)	Relapse (*n*=5)		Abstinent (*n*=6)	Relapse (*n*=6)	
Demographic factors						
Age (years)	44.0±6.0	39.8±9,3	0.37	44.0±5.3	43.3±12.7	0.9
Gender (%)	80 m, 20 f	40 m, 60 f	0.13	83.3 m, 16.7 f	83.3 m, 16.7 f	1.0
Smoking (%)	80	80	1.0	83.3	66.7	0.5
Psychometric characteristics						
Craving at baseline	15.2±13.1	11.9±11.3	0.62	10.0±5.4	10.2±1.8	0.94
Craving post-intervention	10.0±10.0	8.8±8.8	0.85	6.5±4.9	5.5±5.4	1.0
Anxiety at baseline	6.0±3.8	6.2±5.2	0.94	5.8±5.2	3.2±3.2	0.31
Anxiety post-intervention	4.0±3.7	5.1±7.3	1.0	3.8±2.9	3.2±3.9	0.58
Depression at baseline	9.4±7.3	11.3±9.0	0.69	9.5±7.2	8.2±5.8	0.73
Depression post-intervention	5.4±5.1	10.5±10.8	0.37	6.7±5.5	6.5±6.3	0.81
Heart rate variability						
CVNN at baseline (%)	3.5±1.1	5.1±2.1	0.15	3.6±0.7	4.9±1.6	0.11
CVNN post-intervention (%)	4.5±1.21	5.5±1.7	0.27	3.5±1.3	5.4±2.7	0.16
HF at baseline (ms^2^)	222.8±258.8	606.6±1331.2	0.2	193.1±182.5	310.4±312.3	0.81
HF post-intervention (ms^2^)	93.4±101.5	405.9±400.9	0.07	86.2±42.3	1074.8±1589.1	0.31
LF at baseline (ms^2^)	229.9±122.7	681.96±811.4	0.16	260.6±216.0	674.9±665.8	0.18
LF post-intervention (ms^2^)	546.5±409.5	615.82±573.9	0.85	343.9±317.2	1246.2±2444.4	0.81
TP at baseline (ms^2^)	915.8±659.7	2332.2±2475.8	0.16	1255.3±1331.8	1425.4±1248.9	1.0
TP post-intervention (ms^2^)	1138.3±597.7	1945.7±1773.6	0.44	713.9±539.3	2480.8±4048.6	0.39

Analyses revealed no differences between abstinent patients and those who had relapse in any of the included demographic, psychometric or HRV parameters. Among patients who completed follow-up, this was true both in between patients who have undergone HRV-biofeedback one year earlier and those who have received rehabilitation only. “Craving” refers to the Obsessive Compulsive Drinking Scale score. “Anxiety” and “Depression” refer to scores of subscales Anxiety and Depression from the Symptom Checklist-90

*CVNN* coefficient of variation of R-R intervals, *HF* high frequency, *LF* low frequency, *TP* total power

### Logistic regression analyses

None of the assessed demographic and outcome variables were associated with abstinence 1 year after the HRV-biofeedback intervention. (Table 3) The same was true for control patients who underwent rehabilitation care alone. As bivariate models did not reveal any significant associations, no multivariate models were built.

**Table 3:** Bivariate linear regression analyses

	HRV-biofeedback (*n*=15)		Control (*n*=12)	
	Unstandardized B coefficient	*p*- value	Unstandardized B coefficient	*p*-value
Demographic factors				
Age	0.07	0.35	<0.01	0.89
Gender	1.79	0.16	0.00	1.00
Smoking	0.67	0.32	0.20	0.78
Psychometric characteristics				
Craving at baseline	0.02	0.59	-0.01	0.93
Craving post-intervention	0.01	0.79	0.04	0.71
Anxiety at baseline	-0.10	0.93	0.16	0.29
Anxiety post-intervention	-0.03	0.73	0.06	0.79
Depression at baseline	-0.03	0.66	0.03	0.70
Depression post-intervention	-0.08	0.33	>0.01	0.95
Heart rate variability				
CVNN at baseline	-0.65	0.17	-1.07	0.16
CVNN post-intervention	-0.54	0.26	-0,79	0.21
HF at baseline	<-0.01	0.26	<-0.01	0.41
HF post-intervention	<-0.01	0.26	<-0.01	0.30
LF at baseline	<-0.01	0.23	<-0.01	0.22
LF post-intervention	0.00	0.79	<-0.01	0.48
TP at baseline	0	0.46	<0.01	0.75
TP post-intervention	<0.01	0.79	<-0.01	0.32

Regression analyses in patients who completed follow-up did not reveal any predictors of abstinence among demographic, psychometric or HRV parameters both in patients who have been treated with HRV-biofeedback and controls. “Craving” refers to the Obsessive Compulsive Drinking Scale score. “Anxiety” and “Depression” refer to scores of subscales Anxiety and Depression from the Symptom Checklist-90

*CVNN* coefficient of variation of R-R intervals, *HF* high frequency, *LF* low frequency, *TP* total power

## Discussion

In this follow-up study 1 year after a randomized controlled trial of HRV-biofeedback in patients with alcohol addiction, we observed a tendency toward higher rates of long-term abstinence in the interventional study arm when compared with patients of the control arm that have not undergone HRV-biofeedback. Viewed in conjunction with our previous observation of improvement in cardiac autonomic and neurovascular function and reduction of craving and anxiety after HRV-biofeedback in the same study population, these findings warrant follow-up research to confirm this trend in a larger study population and assess the neurophysiological mechanisms whereby HRV-biofeedback might alter long-term abstinence [10]. Although our study was not powered to show significant group differences 1 year post-treatment, our data might contribute to generating the hypothesis that HRV-biofeedback has beneficial effects on long-term abstinence.

A recent randomized clinical trial had confirmed that HRV-biofeedback as adjuvant therapy leads to reduced craving in patients with addiction to alcohol and drugs [12]. Similar to our previous randomized controlled trial, this investigation has been able to show a trend toward improvement of autonomic cardiac function following the intervention. While long-term abstinence has not been assessed in this study, the observed weak short-term effect on HRV might offer an explanation why in our study changes in abstinence 1 year post-intervention did not reach statistical significance. In both studies, the duration and frequency of HRV-biofeedback training was limited, corresponding to 6 treatment sessions over a period of 2 weeks in our RCT and 3 sessions during 3 weeks in the work of Eddie et al. This relatively short treatment regimen might have been insufficient to achieve an improvement in autonomic cardiac function which influences craving, and thereby chance of relapse, to a degree which translates into long-term abstinence beyond the duration of the intervention. Although our RCT showed that at follow up 3 weeks post-intervention, HRV was already decreasing toward baseline, the reduction in craving was sustained until the last follow up 6 weeks post-intervention. In fact, treatment with HRV-biofeedback can improve psychometric measures such as subjective fatigue independent from changes in autonomic cardiac function [13]. Taken together, this might implicate that HRV-biofeedback treatment has to be applied more often and over a longer time period to achieve a sustained effect on craving and thereby abstinence. However, dose-response studies in larger study populations seem necessary to elucidate the therapeutic potential of this treatment to improve outcomes of rehabilitation for alcohol addiction.

Moreover, the underlying mechanism whereby improvement of the autonomic cardiac function leads to alleviation of craving, and possibly changes in abstinence, needs to be elucidated. The observed effect of HRV-biofeedback on autonomic cardiac function and craving might be explained by the physiological mechanism of action of this technique. HRV-biofeedback is a behavioral intervention that targets enhancement of the beat-to-beat fluctuations of the heart rate (HRV) due paced breathing. Physiologically, the heart rate is determined by the intrinsic sinoatrial node discharge rate as well its autonomic alteration mediated by cardiac sympathetic and parasympathetic activity [14]. The preganglionic sympathetic and parasympathetic outflow is determined by the central autonomic network (CAN), a functional unit of the central nerve system which regulates adaptive visceromotor and behavioral responses to internal and environmental stimuli [15]. Interestingly, there is an overlap between the CAN and the anterior executive region (AER), another functional network of brain centers which is responsible of assessing the motivational content of internal and external stimuli and regulating context-dependent behaviors. Cerebral centers that are part of both CAN and AER comprise the insular, anterior cingulate and prefrontal cortices as well as the amygdala and the periaquaductal gray [16]. The functional overlap of these centers viewed in conjunction with the previous observation of reduced HRV in several mental disorders support the concept of HRV constituting an index of individual self-regulation and psychological flexibility [17, 18]. Although the exact neurophysiological interaction between CAN and AER is poorly elucidated, their common capacity of altering HRV might explain why improvement of HRV could also lead to stabilization of craving and anxiety in alcohol dependent patients. Interestingly, decrease in high frequency HRV, a spectral analysis based parameter of parasympathetic activity, has been shown to predict alcohol craving independent of age, anxiety and levels of alcohol consumption. This suggests that impaired cardiac parasympathetic function is associated with increased measures of craving [19]. Furthermore, HRV reactivity to cue-exposure has been pointed as a predictor of relapse after treatment independent of therapeutic regime and after controlling for the severity of the alcohol dependence [20]. In fact, neuroimaging studies showed that patients with alcohol use disorder exposed to alcohol-related cues which induce craving, i.e. alcohol print advertisements or images (or the taste or smell) of their favorite alcoholic beverage, present activation of ventral striatum, anterior cingulate and ventromedial prefrontal cortices [21].

Our study is limited by a low return rate of the mailed questionnaires. Therefore, the observed differences in abstinence rates between HRV-biofeedback treated and control patients might not have reached statistical significance due to a type two error. In the intent-to-treat analysis, statistical power might have been compromised by the conservative approach of imputing missing data, where all patients who have not completed follow-up were considered to have relapsed. This limitation might be overcome by increasing the sample size in future studies. Alternatively, it might be necessary to increase the frequency and duration of the HRV-biofeedback treatment sessions to translate the observed trend into a significant long-term difference.

We did not assess the amount of past-year drinking, therefore we cannot comment on possible associations between treatment with HRV-biofeedback and the severity of relapse. Furthermore, patients weren’t asked to continue paced breathing after the intervention and therefore we haven’t assessed the rate of those still practicing the breathing technique after discharge. This might further explain why in our study observed trends of increase in abstinence have not reached statistical significance.

Taken together, our study forms a basis for a long-term investigation of HRV-biofeedback in patients with alcohol addiction which should include a larger sample size and a dose-response protocol to identify the optimal regimen and achieve a sustained improvement in craving and autonomic function. This, in turn, might translate into improved long-term abstinence.

## Conclusions

Our data suggest that HRV-biofeedback might contribute to long-term abstinence when applied in addition to rehabilitation care. Since the trends observed in this follow-up study did not reach statistical significance, further research is warranted to confirm this hypothesis in a larger study population.

## Abbreviations

AER: Anterior executive region
CAN: Central autonomic network
CVNN: Coefficient of variation of R-R intervals
f: female
HF: High frequency
HRV: Heart rate variability
LF: Low frequency
m: male
ns: not significant
RCT: Randomized controlled trial
SD: Standard deviation
TP: Total power
vs: versus
